# Neonate, Infant, and Child Mortality in North Africa and Middle East by Cause: An Analysis for the Global Burden of Disease Study 2019

**DOI:** 10.34172/aim.2022.122

**Published:** 2022-12-01

**Authors:** Sadaf G. Sepanlou, Hossein Rezaei Aliabadi, Reza Malekzadeh, Mohsen Naghavi, GBD Child Mortality in Middle East Collaborators

**Affiliations:** ^1^Digestive Disease Research Institute, Tehran University of Medical Sciences, Tehran, Iran; ^2^Bam University of Medical Sciences, Bam, Iran; ^3^Institute for Health Metrics and Evaluation, School of Medicine, University of Washington, Seattle, USA

**Keywords:** Child mortality, Inequality, Infant mortality, Middle East, North Africa

## Abstract

**Background:**

During the past three decades, neonate, infant, and child mortality declined in North Africa and Middle East. However, there is substantial heterogeneity in mortality rates across countries.

**Methods:**

This study is part of the Global Burden of Diseases study (GBD) 2019. We report the number as well as mortality rates for neonates, infants, and children by cause across 21 countries in the region since 1990.

**Results:**

Between 1990 and 2019, the neonate mortality rate in the region declined from 31.9 (29.8, 34.0) to 12.2 (11.1, 13.3) per 1000 live births. Respective figures for under 5 mortality rates (U5MRs) were 79.1 (75.7, 82.7) in 1990 and 24.4 (22.3, 26.7) per 1000 live births in 2019. The majority of deaths among children under 5 years were due to under 1 year deaths: 75.9% in 1990 and 81.8% in 2019. Mortality rates in males were higher than females. The mortality rate among neonates ranged from 2.4 (2.1, 2.6) per 1000 live births in Bahrain to 25.0 (21.6, 28.4) in Afghanistan in 2019. Similarly, in 2019, the U5MR ranged from 5.0 (4.2–6.0) per 1000 live births in United Arab Emirates to 55.3 (47.9–63.5) in Afghanistan. Neonatal disorders, congenital birth defects, and lower respiratory infections were the three main causes of neonate, infant, and child mortality in almost all countries in the region.

**Conclusion:**

In 2019, most countries in this region have achieved the SDG targets for neonate and child mortality. However, there is still substantial heterogeneity across countries.

## Introduction

 During the past three decades, the world witnessed substantial reduction in neonate, infant, and child mortality. The global number of deaths among children under 5 years old decreased by 58%, from 12 million in 1990 to slightly over 5 million in 2019. Under 5 mortality rate (U5MR) declined from 87 per 1000 live births in 1990 to 37 per 1000 live births in 2019.^1,2^ However, the decline hasn’t been homogenous across regions and nations with various levels of socioeconomic development, and the existing disparity is not negligible.^3,4^ In 2019, more than half of the global deaths among children under 5 years old occurred in low-income countries, while high-income nations accounted for merely 4 percent of total deaths.^1-3,5-7^ If this trend continues, many low-income nations will be unable to achieve the targets set out in the sustainable development goals (SDGs) for neonate and child mortality by 2030.^8,9^

 Similar trend in neonate, infant, and child mortality and the accompanying heterogeneity is observed in North Africa and Middle East. This region is unique in many ways. Islam is the prominent religion, and the region is comprised of 21 countries that substantially vary in their socioeconomic development and the infrastructure of their health systems. Ultimately, the region is afflicted by ongoing conflicts, and political, economic, and social turmoil that affects the health of vulnerable groups, mainly mothers, neonates, infants, and children.^10^ In spite of the progress in children’s health during the past 30 years, there are still substantial disparities between countries in the region. In this paper we present the burden of neonate, infant, and child mortality by cause in North Africa and Middle East 1990 to 2019.

## Materials and Methods

 This study is part of GBD 2019, which was a systematic effort to estimate the levels, trends, and causes of mortality by sex, age, year (1990 to 2019), and location. In this article we report the estimates for neonate (0-27 days), infant (under 1 year), and child (under 5 year) mortality by cause at the regional and national levels in north Africa and Middle East and across 21 countries in this region including: Afghanistan, Algeria, Bahrain, Egypt, Iran, Iraq, Jordan, Kuwait, Lebanon, Libya, Morocco, Oman, Palestine, Qatar, Saudi Arabia, Sudan, Syrian Arab Republic, Tunisia, Turkey, United Arab Emirates, and Yemen.

###  All-Cause Mortality

 We used multiple data types to estimate all-cause mortality rate, including data from vital registration system, and surveys and censuses, from which we extracted household death recall data, summary birth histories, and complete birth histories.

 First, the ratio of children ever surviving to children ever born by age of mother was converted to an estimate of U5MR. Second, a multi-stage process was used to include a non-linear mixed effects model, source bias correction, spatiotemporal smoothing, and Gaussian process regression to synthesize U5MR data into both sex U5MR estimates for every location-year in North Africa and Middle East. Complete time series of lag-distributed income, maternal education (mean years of education among women of reproductive age, 15–49 years), and childhood HIV crude were incorporated into the mixed effects model. Third, analogous multi-stage location-year specific models were used to estimate the sex-ratio of under-5 mortality and age- and sex specific probability of death for neonates and infants. Fourth, death counts were calculated by simulating exposure to the estimated age-specific mortality probabilities for weekly birth cohorts from GBD 2019 live birth estimates. Fifth, death counts were divided by corresponding age-specific mortality rates (deaths per person-year) which is the version of the mortality metric presented in most GBD publications and visualization tools. Finally, fatal discontinuities, defined as sharp change in mortality by year resulting from unexpected events such as natural disasters and conflict, were estimated separately and appended to generate final all-cause mortality estimates.^11^

###  Cause-Specific Mortality

 For calculating cause specific mortality, cause of death data from above mentioned data sources were in the first step mapped to the GBD cause list using various revisions of the International Classification of Diseases and Injuries.^1^ Second, data was corrected for misclassification to non-specific codes, impossible causes, or clinical syndromes by redistributing these so-called “garbage codes” to the most likely underlying causes of death by age, sex, location, and year using a number of previously described statistical approaches.^1,11^ Third, a non-zero floor was calculated defined as + /- 2 median absolute deviations for each age-sex-cause combination to reduce downward bias that could be introduced by redistribution for comparatively rare causes in locations with good data quality. Fourth, a Bayesian noise-reduction algorithm was applied based on a Poisson count model to account for stochastic variation in data and reduce the upward bias that could be introduced by zero counts.^1,11^

 After completion of data processing, a complete time series of mortality estimates was generated for each cause. Cause of Death Ensemble modeling (CODEm) was the most commonly-used modeling platform. CODEm uses a train-test-test approach to evaluate multiple submodels, each of which contain different combinations of candidate covariates, to systematically maximize out of sample predictive validity and weight them accordingly in generating a final ensemble model.^12^ Cause-specific mortality estimates were then scaled to match all-cause mortality estimates for each age group, location, sex, and year in a process called CoDCorrect.^1^ Similar to the all-cause mortality analysis above, cause-specific deaths due to fatal discontinuities (eg, conflict and terrorism, major transportation accidents, natural disasters, epidemics, other forms of disaster such as large fires or the collapse of large buildings) were estimated separately using vital registration and alternative databases and appended to final results. Cause-specific mortality rates were converted into probabilities by multiplying all-cause probability of death by cause fraction.

###  Disparity in Mortality

 We used the socio-demographic index (SDI) to determine the relationship between the development level of a country and infant and child mortality. SDI is a composite indicator of a country’s lag-distributed income per capita, average years of schooling among individuals over 15 years old, and the fertility rate in females under the age of 25 years.^2^

 The methods we used for propagating uncertainty are the same as those used in previous GBD iterations.^1,5^ All all-cause and cause-specific mortality estimation components are based on 1000 draws, or simulations, by age, sex, location, and year. Point estimates were derived from the mean of the draws, and 95% uncertainty intervals (UIs) were calculated as the 2.5^th^ and 97.5^th^ percentiles of the draws.

###  Role of the Funding Source 

 The funders of this study had no role in study design, data collection, data analysis, data interpretation, or the writing of the report. The corresponding author had full access to the data in the study and final responsibility for the decision to submit for publication.

## Results

**Figure 1:**
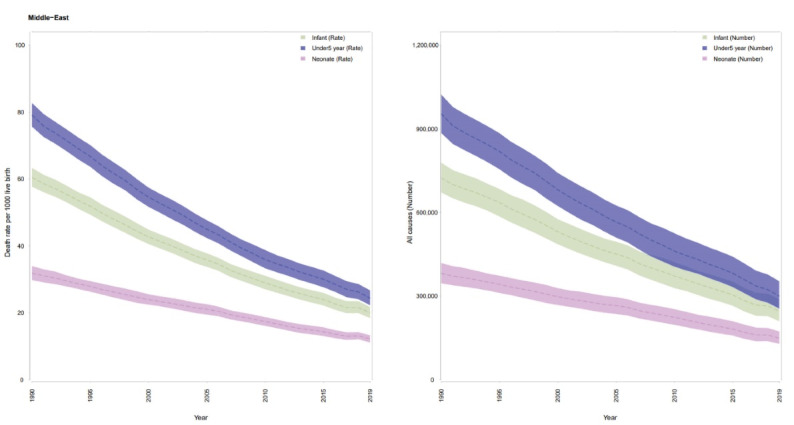
The Trend in Neonate, Infant, and Child Mortality Counts and Rates at Regional Level From 1990 to 2019.

 In 1990, a total of 381 311 (95% UI: 346 099, 418 595) deaths occurred among neonates less than 28 days in north Africa and Middle East, which steadily decreased to 149 527 (128 847, 172 959) deaths in 2019. The rate of neonatal mortality decreased as well from 31.9 (29.8, 34,0) per 1000 live births in 1990 to 12.2 (11.1, 13.3) per 1000 live births in 2019 (Figure 1, Table 1).

**Table 1:** Neonatal Deaths and Neonatal Mortality Per 1000 Live Births in 1990 and 2019 and the Percent Change in Mortality Rate from 1990 to 2019 by Country.

**Location**	**Count 1990**	**Rate 1990**	**Count 2019**	**Rate 2019**	**Percent Change**
North Africa and Middle East	381311 (346099, 418595)	31.9 (29.8, 34)	149527 (128847, 172959)	12.2 (11.1, 13.3)	-61.7
Afghanistan	25428 (21382, 29480)	48.2 (40.8, 55.8)	37406 (31255, 44206)	25 (21.6, 28.4)	-48.2
Algeria	19948 (16488, 23577)	25.9 (22, 30)	10651 (8216, 13410)	12 (9.8, 14.5)	-53.6
Bahrain	160 (138, 182)	11.6 (10.4, 12.8)	31 (25, 39)	2.4 (2.1, 2.6)	-79.7
Egypt	61991 (51399, 73280)	31.7 (26.4, 36.9)	11756 (8246, 16207)	5.6 (4.2, 7.2)	-82.5
Iran (Islamic Republic of)	55513 (46255, 65088)	31.8 (28.1, 35.5)	9145 (7444, 11136)	6.8 (6.1, 7.4)	-78.7
Iraq	19488 (17186, 21933)	26.6 (23.6, 29.9)	9132 (6614, 12751)	9.5 (7.9, 11.5)	-64.3
Jordan	2615 (2335, 2923)	19 (17.3, 20.9)	2127 (1565, 2932)	8.8 (7.5, 10.6)	-53.7
Kuwait	401 (362, 445)	10.1 (9.2, 11)	310 (242, 402)	5.1 (4.3, 6)	-49.6
Lebanon	2012 (1630, 2418)	17.7 (15, 20.5)	521 (373, 734)	4.8 (4, 5.9)	-72.8
Libya	3418 (2886, 3975)	21.9 (19, 25.2)	458 (351, 583)	5.6 (4.7, 6.7)	-74.3
Morocco	28496 (24391, 32923)	35.3 (31.6, 38.9)	6757 (5125, 8703)	11.1 (9.7, 12.5)	-68.7
Oman	1173 (965, 1428)	17.7 (14.9, 21.1)	418 (357, 482)	5.4 (4.9, 5.9)	-69.6
Palestine	1737 (1459, 2005)	18.1 (15.6, 20.7)	800 (606, 1061)	6.4 (5.4, 7.6)	-64.9
Qatar	146 (117, 177)	13.8 (11.5, 16.2)	113 (91, 144)	4.2 (3.5, 5.1)	-69.4
Saudi Arabia	9672 (7461, 12304)	17.9 (14.3, 22)	1202 (950, 1519)	2.6 (2.2, 3.2)	-85.3
Sudan	49396 (42516, 56378)	46.9 (41.4, 52.3)	25701 (20601, 32481)	21.3 (18.9, 24.1)	-54.6
Syrian Arab Republic	12436 (10059, 15136)	24.1 (19.6, 29.1)	1590 (1316, 1902)	6.9 (5.7, 8.3)	-71.4
Tunisia	5914 (4745, 7332)	24.5 (20, 29.3)	1144 (903, 1421)	6.8 (5.7, 8.1)	-72.2
Turkey	52158 (44638, 60983)	30.9 (27, 35.2)	8384 (6707, 10429)	8.5 (7.2, 10.2)	-72.4
United Arab Emirates	526 (443, 622)	11.3 (9.8, 13)	145 (109, 197)	2.6 (2.2, 3.1)	-77.2
Yemen	28427 (23995, 33380)	41.7 (35.1, 49)	21585 (17661, 26000)	22.8 (18.8, 27.4)	-45.3

 In 1990, a total of 723 860 (672 030, 778 850) deaths occurred among infants under 1 year in north Africa and Middle East, which decreased to 245450 (209700, 288510) deaths in 2019. The rate of infant mortality decreased as well from 60.4 (57.7, 63.3) per 1000 live births in 1990 to 20 (18.4, 21.8) per 1000 live births in 2019 (Figure 1, Table 2).

**Table 2:** Infant Deaths and Infant Mortality Per 1000 Live Births in 1990 and 2019 and the Percent Change in Mortality Rate from 1990 to 2019 by Country

**Location**	**Count 1990**	**Rate 1990**	**Count 2019**	**Rate 2019**	**Percent Change**
North Africa and Middle East	723859 (672030, 778848)	60.4 (57.7, 63.3)	245451 (209701, 288513)	20 (18.4, 21.8)	-66.9
Afghanistan	56358 (48541, 64039)	107.8 (93.1, 122.3)	67898 (58145, 78732)	45.7 (40.8, 50.6)	-57.7
Algeria	39149 (33860, 44986)	50.8 (45.7, 56.1)	15201 (12649, 18065)	17.1 (15, 19.6)	-66.2
Bahrain	279 (254, 305)	20.3 (19.1, 21.5)	69 (54, 86)	5.2 (4.6, 5.9)	-74.3
Egypt	122205 (108178, 137480)	62.4 (55.6, 69.7)	26026 (19484, 34243)	12.3 (10.3, 14.7)	-80.3
Iran (Islamic Republic of)	96630 (81362, 112155)	54.9 (48.7, 61.3)	12937 (10548, 15718)	9.5 (8.6, 10.5)	-82.6
Iraq	33694 (29756, 37685)	45.9 (40.9, 51.2)	11943 (8662, 16673)	12.4 (10.4, 15.1)	-72.9
Jordan	4061 (3630, 4535)	29.6 (26.8, 32.9)	3165 (2329, 4365)	13.2 (11.2, 15.8)	-55.5
Kuwait	634 (572, 703)	15.6 (14.2, 17)	469 (370, 605)	7.7 (6.5, 9.1)	-50.5
Lebanon	3133 (2692, 3606)	27.5 (24.3, 31)	842 (605, 1182)	7.8 (6.5, 9.5)	-71.8
Libya	5418 (4595, 6310)	34.7 (29.8, 39.8)	669 (514, 851)	8.2 (6.9, 9.7)	-76.4
Morocco	49731 (43578, 56329)	61.7 (56.6, 66.6)	9590 (6766, 13353)	15.6 (13.1, 18.7)	-74.7
Oman	1835 (1509, 2230)	27.6 (23.4, 32.9)	646 (550, 746)	8.3 (7.5, 9.1)	-70
Palestine	3069 (2755, 3412)	32.2 (29.1, 35.7)	1288 (974, 1711)	10.2 (8.7, 12.3)	-68.2
Qatar	235 (190, 285)	22.1 (18.7, 25.9)	178 (143, 227)	6.7 (5.6, 8.1)	-69.9
Saudi Arabia	18752 (14922, 22964)	34.8 (29.2, 40.8)	2031 (1608, 2561)	4.5 (3.7, 5.5)	-87.2
Sudan	91642 (82425, 101270)	87.6 (79.8, 95.5)	40090 (29616, 54405)	33.1 (28.2, 39.4)	-62.2
Syrian Arab Republic	21094 (18430, 23824)	41 (36.2, 46.2)	2194 (1738, 2744)	9.5 (8.1, 11)	-76.9
Tunisia	10334 (8882, 11968)	42.6 (38.3, 47.5)	1578 (1246, 1958)	9.4 (7.9, 11.2)	-78
Turkey	99030 (87611, 111003)	58.6 (53.6, 64.3)	12164 (9733, 15123)	12.4 (10.4, 14.8)	-78.9
United Arab Emirates	779 (666, 910)	16.7 (14.7, 19.2)	217 (163, 294)	3.8 (3.2, 4.6)	-77.3
Yemen	65310 (59386, 71936)	96.5 (88.2, 105.6)	36006 (29659, 43509)	38.1 (32.5, 44.6)	-60.5

 Respective figures for under 5 mortality show similar decline. In 1990 a total of 954 160 (885 050, 1 023 530) deaths occurred among children under 5 years in north Africa and Middle East, which declined to 300 000 (255 260, 353 290) deaths in 2019. Respective figures for U5MRs were 79.1 (75.7, 82.7) in 1990 and 24.4 (22.3, 26.7) per 1000 live births in 2019 (Figure 1, Table 3). The majority of deaths among children under 5 years were due to under 1 year deaths: 75.9% in 1990 and 81.8% in 2019.

**Table 3:** Child Deaths and Child Mortality Per 1000 Live Births in 1990 and 2019 and the Percent Change in Mortality Rate from 1990 to 2019 by Country

**Location**	**Count 1990**	**Rate 1990**	**Count 2019**	**Rate 2019**	**Percent Change**
North Africa and Middle East	954160 (885051, 1023528)	79.1 (75.7, 82.7)	300003 (255260, 353292)	24.4 (22.3, 26.7)	-69.2
Afghanistan	78170 (70157, 86995)	151.1 (136.7, 167.4)	81352 (67932, 97234)	55.3 (47.9, 63.5)	-63.4
Algeria	45891 (40132, 52202)	59.5 (54.4, 65.1)	17319 (14466, 20537)	19.5 (17, 22.4)	-67.2
Bahrain	316 (287, 345)	23.1 (22, 24.3)	87 (70, 109)	6.5 (5.8, 7.4)	-71.8
Egypt	164220 (148155, 181014)	83.5 (76, 91.2)	32602 (24627, 42641)	15.3 (12.8, 18.3)	-81.7
Iran (Islamic Republic of)	127608 (107686, 147945)	71.2 (63.6, 79.1)	15231 (12690, 18363)	11.1 (10.2, 12)	-84.4
Iraq	40246 (36130, 44178)	54.7 (50.1, 59.8)	15008 (11028, 20740)	15.7 (13.2, 18.9)	-71.4
Jordan	4604 (4125, 5109)	33.7 (30.8, 37.1)	3640 (2682, 5006)	15.3 (13, 18.3)	-54.7
Kuwait	890 (814, 970)	21.2 (19.8, 22.5)	555 (440, 708)	9.2 (7.8, 10.8)	-56.6
Lebanon	3606 (3085, 4170)	31.8 (27.8, 36)	983 (708, 1378)	9 (7.5, 11)	-71.7
Libya	6786 (5729, 7922)	43.4 (37.7, 49.9)	1109 (869, 1390)	13.3 (11.3, 15.7)	-69.3
Morocco	60435 (52488, 69442)	75.1 (68.3, 81.9)	11105 (7855, 15397)	17.9 (15, 21.4)	-76.1
Oman	2343 (1937, 2848)	35.3 (30.1, 42)	809 (690, 937)	10.4 (9.4, 11.4)	-70.6
Palestine	4016 (3642, 4435)	43 (39.4, 47.2)	1562 (1181, 2064)	12.4 (10.5, 14.9)	-71.2
Qatar	279 (225, 339)	26.3 (22.1, 30.7)	214 (172, 274)	8 (6.7, 9.7)	-69.5
Saudi Arabia	24026 (19470, 29118)	44.9 (38.2, 52.2)	2614 (2069, 3305)	5.7 (4.8, 7)	-87.2
Sudan	145281 (131797, 159313)	142.2 (131.8, 153.7)	50708 (37299, 68604)	41.9 (35.7, 50)	-70.5
Syrian Arab Republic	25521 (22218, 28925)	49.9 (43.8, 56.3)	3211 (2564, 3994)	13.6 (11.7, 15.8)	-72.8
Tunisia	12337 (10551, 14306)	50.7 (45.6, 56.4)	1923 (1526, 2380)	11.3 (9.5, 13.5)	-77.7
Turkey	118642 (104664, 134173)	70.2 (63.9, 77.1)	15133 (12148, 18731)	15.4 (12.9, 18.4)	-78.1
United Arab Emirates	1004 (868, 1158)	21.6 (19.2, 24.3)	295 (220, 399)	5 (4.2, 6)	-77
Yemen	87296 (79858, 94993)	130.6 (120.4, 140.9)	44236 (36660, 53124)	46.7 (40.2, 54.4)	-64.2

**Figure 2:**
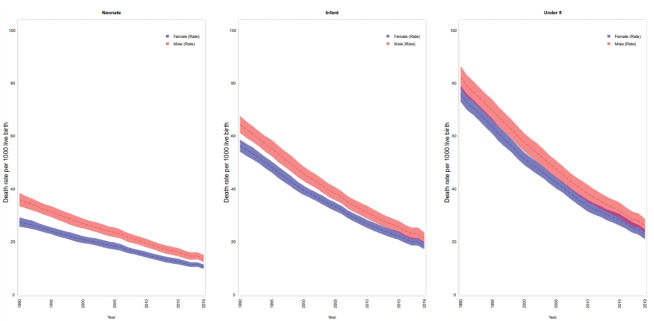
Neonatal, infant, and child mortality rates by sex in north Africa and Middle East from 1990 to 2019.

Figure 2 demonstrates that neonatal, infant, and child mortality rates were higher in males than females in all years from 1990 to 2019 in North Africa and Middle East. The same pattern was observed in all countries.

**Figure 3:**
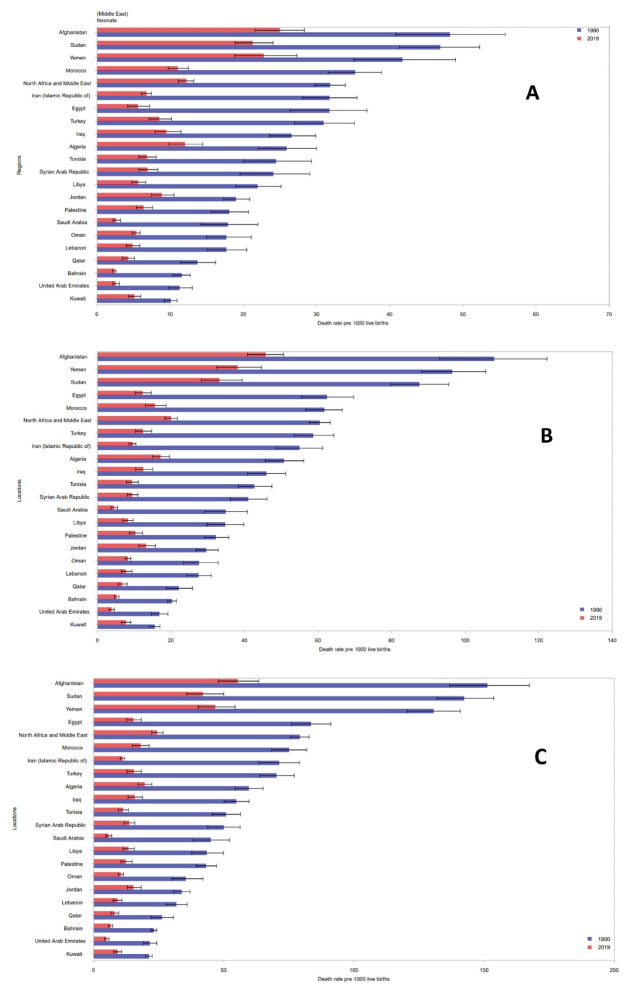
Neonate, Infant, and Child Mortality Rates Across Countries in the Region in 1990 and 2019. A() Neonate Mortality. (B) Infant mortality. (C) Child mortality.

 The mortality rate among neonates ranged from 2.4 (2.1, 2.6) per 1000 live births in Bahrain to 21.3 (18.9, 24.1) in Sudan, 22.8 (18.8, 27.4) in Yemen, and 25 (21.6, 28.4) in Afghanistan in 2019. The highest decline in neonatal mortality between 1990 and 2019 was observed in Saudi Arabia (-85.3%) and the lowest decline was estimated for Yemen (-45.3%) (Figure 3A, Table 1). In 2019, all countries in North Africa and Middle East achieved the SDG target of neonatal mortality less than 12 deaths per 1000 live births except for the three countries of Afghanistan, Yemen, and Sudan.

 The mortality rate among infants under 1 year ranged from 3.8 (3.2, 4.6) per 1000 live births in the United Arab Emirates to 33.1 (28.2, 39.4) in Sudan, 38.1 (32.5, 44.6) in Yemen, and 45.7 (40.8, 50.6) in Afghanistan in 2019. The highest decline in infant mortality between 1990 and 2019 was observed in Saudi Arabia (-87.2%) and the lowest decline was estimated for Kuwait (-50.5%) (Figure 3B, Table 2).

 Similarly, in 2019, the U5MR ranged from 5.0 (4.2–6.0) per 1000 live births in the United Arab Emirates to 41.9 (35.7, 50) in Sudan, 46.7 (40.2, 54.4) in Yemen, and 55.3 (47.9–63.5) in Afghanistan. Actually in 2019, all countries in North Africa and Middle East achieved the SDG target of under 5 mortality less than 25 deaths per 1000 live births except for the three countries of Afghanistan, Yemen, and Sudan. The highest decline in under 5 mortality since 1990 was observed in Saudi Arabia (-87.2%) and the lowest (-54.7%) in Jordan (Figure 3C, Table 3).

**Figure 4:**
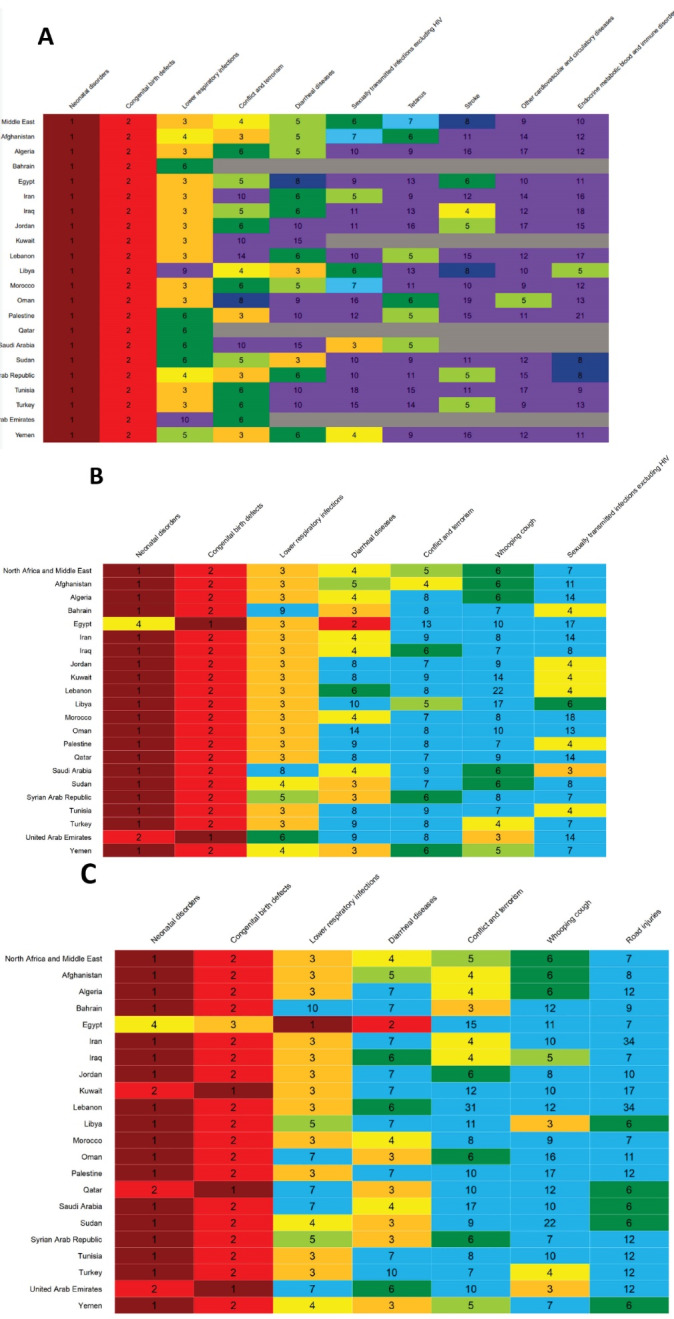
Infant and Child Deaths by Cause in 2019. (A) Neonate mortality. (B) Infant mortality. (C) Child mortality.

 Neonatal disorders, congenital birth defects, and lower respiratory infections were the three main causes of neonatal, infant, and child mortality in almost all countries in North Africa and Middle East (Figure 4A to 4C). Egypt was an exception in which, diarrheal diseases ranked first in both infant and child mortality (Figure 4B and 4C). Conflict and terrorism, diarrheal diseases, sexually transmitted infections, and tetanus ranked 4^th^ to 7^th^ among neonates (Figure 4A). Diarrheal diseases, conflict and terrorism, and whooping cough ranked 4^th^ to 6^th^ in both infants and children in most countries (Figure 4B and 4C). In infants, sexually transmitted infections excluding HIV ranked 7^th^ in 2019. In children, however, road injuries ranked 7^th^.

 In North Africa and Middle East, the share of neonatal disorders out of all causes of neonatal mortality was 65.1% in 1990 and 64.7% in 2019. Among all neonatal disorders in 2019, 55.2% were due to neonatal preterm birth, 27.9% were due to other neonatal disorders, 10% were due to neonatal encephalopathy due to birth asphyxia and trauma, 5.5% were due to neonatal sepsis and other neonatal infections, and 1.3% were due to hemolytic disease and other neonatal jaundice. The order and share of each cause out of neonatal diseases were almost the same in 1990.

 The share of neonatal disorders out of all causes of infant mortality was 45.0% in 2019. Respective figures for congenital birth defects and lower respiratory infections were 22.6% and 9.9% in 2019 (Figure 4B). In children, however, the share of neonatal disorders out of the total causes of death among children under 5 years was 37.3%. Respective figures for congenital birth defects and lower respiratory infections were 21.0% and 10.2% in 2019.

**Figure 5:**
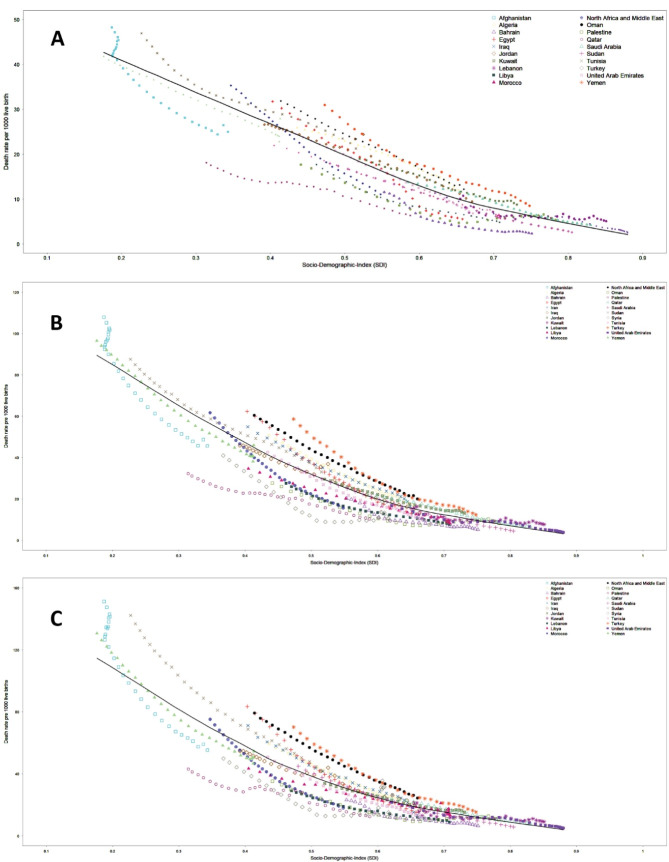
The Trend in Infant and Child Mortality Along with Increase in Sociodemographic Index Across Countries in North Africa and Middle East from 1990 to 2019. (A) Neonate Mortality. (B) Infant mortality. (C) Child mortality.

Figure 5 demonstrates the trends in neonate, infant, and child mortality versus SDI across countries from 1990 to 2019. Trends demonstrate an apparent decrease in mortality in all countries. The ratio of highest to lowest death rates in neonates, however, increased from 4.8 in 1990 to 10.4 in 2019. Respective figures for infant mortality were 6.9 and 12.0 in 1990 and 2019, and for child mortality were 7.1 in 1990 and 11.1 in 2019.

## Discussion

 Our study shows that neonates, infant, and child mortality rates in North Africa and Middle East significantly declined since 1990, by 61.7%, 66.9%, and 69.2% respectively. The number of deaths also shows a significant decrease. Mortality rates for neonates, infants, and children in the region were consistently lower than global estimates during the past three decades. The majority of deaths among children were due to neonate and infant mortality in all years.

 Although the decline is observed in all countries of the region, considerable disparity still exists between high-income and low-income countries. In 2019, the ratio of highest to lowest death rates were 10.4, 12.0, and 11.1 for neonate, infant, and child mortality respectively. Overall, in 2019, Afghanistan, Sudan, and Yemen, which had the lowest SDI in the region, together accounted for 56.6%, 58.7% and 58.8% of total deaths among neonates and infants and children respectively at the regional level and are the only countries that haven’t achieved the SDG targets of less than 12 deaths per 1000 live births for neonates and 25 deaths per 1000 live births for children. High-income Arab countries (Bahrain, Kuwait, Oman, Qatar, Saudi Arabia, and United Arab Emirates) contributed to slightly over 1.5% of total deaths among infants and children in the region. Saudi Arabia had relatively higher mortality rates than other high-income Arab countries in the region in 2019, despite the substantial decrease since 1990. Generally, in countries with SDI lower than the median in the region, neonate, infant, and child mortality rates were higher. Exceptions were Jordan and Turkey, which despite their relatively high SDI had high mortality rates. Palestine and Syrian Arab Republic were the opposite and had low mortality rates despite their low SDI.

 Our study shows that neonatal disorders are the main causes of death among neonates and infants and children in North Africa and Middle East, followed by congenital birth defects, lower respiratory infections, and diarrheal diseases. The pattern is quite similar between countries in this region and between the region and the globe. The age distribution of mortality among children shows that the risk of mortality is highest during the first 28 days of life, the neonatal period.^13^ The high share of neonatal disorders out of under 5 deaths highlights the need for urgent actions in order to save newborn lives. Reduction in neonatal mortality requires greater investment in the healthcare infrastructure with the aim of improving coverage, quality, and equity of care in antenatal period.^14^ It will also require intensive care for mothers and newborns at birth and in the first week of life, which not only prevents neonatal mortality, it will also prevent stillbirths and disability.^15,16^

 Infectious diseases remain the leading causes of infant mortality along with preterm birth and intrapartum-related complications. Moreover, malnutrition in children, particularly in case of severe acute malnutrition, leads to a higher risk of death from these common diseases.^17^ Access to basic interventions such as antenatal care, childbirth delivery care, postnatal care, vaccination, and early childhood care can substantially prevent neonate, infant, and child mortality.^17,18^ Reduction in infectious causes of neonate, infant, and child mortality requires improved nutrition, extensive vaccination programs, and improved quality of care.^17^ As infectious diseases disproportionately affect children in poor settings and remain highly prevalent, integrated and targeted policies are mandatory.^19^ Addressing diarrheal diseases requires improved sanitation and hygiene, provision of safe drinking water, vaccination, and oral rehydration therapy.^20^ Preventing neonatal mortality will not be accomplished without skilled personnel attendance at birth and availability of hospital care in case of emergencies.^21^

 The impact of intensive focus and targeted actions of the global community on reductions in child mortality in the past three decades is undeniable. However, progress will not last without the continued, determined, and cooperative action of the global community. Neonate, infant, and child mortality are absolutely preventable.^17^ Equity in access to appropriate high quality health care is key for preventing infant and child mortality specifically in vulnerable groups.

 Our study demonstrates that despite ongoing conflicts, wars, and political and economic turmoil, the sociodemographic index has increased and neonate and infant and child health has improved during the past 30 years in all countries of the region.^22^ However, the pace of improvement is not homogenous across countries. Conflict and terrorism were main causes of infant and child mortality in Afghanistan, Libya, and Syrian Arab Republic in 2019.

 Reliable estimates of neonate, infant, and child mortality at the regional, national, and even subnational levels are essential for evidence-based policymaking to improve children’s survival. In the absence of reliable and continually collected vital registration data, evidence-based estimation of child mortality for monitoring child mortality levels and progress will not be feasible. Nations should be encouraged to improve the quality and completeness of their vital registration systems as the main sources of data for cost-effective policy making at the regional and national levels. Successful experiences can be shared with countries in which sufficient progress in the trend of child mortality has not been achieved.

 The current study is the most comprehensive effort so far that addresses neonate and infant and child mortality by cause and by country in North Africa and Middle East during the past three decades. Yet, our study has limitations similar to previous iterations of GBD, which mainly include lack of primary data. The incompleteness and misclassification of the vital registration data in the region is one of the main limitations of this study. Another limitation of this study is the information bias in recorded data. The bias may be due to the fact that mothers may not exactly remember the date of birth and death of their children. The misclassification in vital registration systems may have had impacts on accuracy of cause of death estimations among infants and children.

 In conclusion, despite considerable improvement in neonate, infant, and child health in North Africa and Middle East, a number of countries have not achieved the SDG targets yet and if the current trend continues, they may be unable to reach the targets by 2030. More importantly, the disparity between countries in the region is increasing. Although the socioeconomic status is a major determinant of children’s health, there are other drivers such as the integrity, quality, and the accessibility of health care system and on the other hand, the socio-political security that determine children’s health. Countries and the international community should take immediate action to end war and bring peace and stability to the region to ensure the health of children.
